# RETRACTION: Impacts of Enhanced Recovery After Surgery Nursing Interventions on Wound Infection and Complications Following Bladder Cancer Surgery: A Meta‐Analysis

**DOI:** 10.1111/iwj.70449

**Published:** 2025-03-26

## Abstract

**RETRACTION:** YangF. , NieJ. , XiaoF. , and LiuJ. , “Impacts of Enhanced Recovery After Surgery Nursing Interventions on Wound Infection and Complications Following Bladder Cancer Surgery: A Meta‐Analysis,” International Wound Journal 21, no. 4 (2024): e14781, 10.1111/iwj.14781.38531376 PMC10965273

The above article, published online on 26 March 2024, in Wiley Online Library (http://onlinelibrary.wiley.com/), has been retracted by agreement between the journal Editor in Chief, Professor Keith Harding; and John Wiley & Sons Ltd. Following an investigation by the publisher, all parties have concluded that this article was accepted solely on the basis of a compromised peer review process. The editors have therefore decided to retract the article. The authors did not respond to our notice regarding the retraction.

**RETRACTION:** F. Yang , J. Nie , F. Xiao , and J. Liu , “Impacts of Enhanced Recovery After Surgery Nursing Interventions on Wound Infection and Complications Following Bladder Cancer Surgery: A Meta‐Analysis,” International Wound Journal 21, no. 4 (2024): e14781, 10.1111/iwj.14781.38531376 PMC10965273

The above article, published online on 26 March 2024, in Wiley Online Library (http://onlinelibrary.wiley.com/), has been retracted by agreement between the journal Editor in Chief, Professor Keith Harding; and John Wiley & Sons Ltd. Following an investigation by the publisher, all parties have concluded that this article was accepted solely on the basis of a compromised peer review process. The editors have therefore decided to retract the article. The authors did not respond to our notice regarding the retraction.

